# Age-Related Differences in Plasma Proteins: How Plasma Proteins Change from Neonates to Adults

**DOI:** 10.1371/journal.pone.0017213

**Published:** 2011-02-18

**Authors:** Vera Ignjatovic, Cera Lai, Robyn Summerhayes, Ulrike Mathesius, Sherif Tawfilis, Matthew A. Perugini, Paul Monagle

**Affiliations:** 1 Murdoch Childrens Research Institute, Royal Children's Hospital, Parkville, Victoria, Australia; 2 Department of Paediatrics, The University of Melbourne, Royal Children's Hospital, Parkville, Victoria, Australia; 3 Research School of Biology, Australian National University, Canberra, Australian Capital Territory, Australia; 4 GE Healthcare Life Sciences Australia, Bundoora, Victoria, Australia; 5 Department of Biochemistry and Molecular Biology, Bio21 Molecular Science and Biotechnology Institute, The University of Melbourne, Parkville, Victoria, Australia; 6 Department of Clinical Haematology, Royal Children's Hospital, Parkville, Victoria, Australia; University of South Florida College of Medicine, United States of America

## Abstract

The incidence of major diseases such as cardiovascular disease, thrombosis and cancer increases with age and is the major cause of mortality world-wide, with neonates and children somehow protected from such diseases of ageing. We hypothesized that there are major developmental differences in plasma proteins and that these contribute to age-related changes in the incidence of major diseases. We evaluated the human plasma proteome in healthy neonates, children and adults using the 2D-DIGE approach. We demonstrate significant changes in number and abundance of up to 100 protein spots that have marked differences in during the transition of the plasma proteome from neonate and child through to adult. These proteins are known to be involved in numerous physiological processes such as iron transport and homeostasis, immune response, haemostasis and apoptosis, amongst others. Importantly, we determined that the proteins that are differentially expressed with age are not the same proteins that are differentially expressed with gender and that the degree of phosphorylation of plasma proteins also changes with age. Given the multi-functionality of these proteins in human physiology, understanding the differences in the plasma proteome in neonates and children compared to adults will make a major contribution to our understanding of developmental biology in humans.

## Introduction

Plasma is a complex biological system and the plasma proteome contains proteins from a variety of cellular localizations, including intracellular and membrane proteins secreted in plasma as a result of cell lysis and cellular turn-over [1]. This system is considered the most informative proteome by clinicians, because it communicates with most cells in the body and hence many disease states tend to be reflected by changes in plasma proteins. Proteomic studies to date have focused on investigation of biomarkers, as well as determining the effects of drugs on various proteomes. The majority of such studies have focused on adults, with very limited investigation of the proteome in neonatal and paediatric population. In children, proteomics has been used to examine changes in expression levels of proteins that occur during Cardiopulmonary Bypass Surgery [2]. A specific approach of proteomics, that uses two-dimensional (2D) gel technology has been used in the paediatric setting, to investigate a number of disease states including: blood disorders [3], sickle cell disease [4], leukemia [5] and cystic fibrosis [6].

The protein diversity of the human plasma proteome has been demonstrated in adults [7], as well as in fetuses, newborns and children [8]. However, the proteins in question were not directly identified and instead, the identity of each protein was based on the position of the protein within the 2-D gel and previously identified proteins from adult samples.

Human Proteome Organization (HUPO) Plasma Proteome Project (PPP) recognizes the importance of analyzing and understanding age-related differences in the plasma proteome by identifying this as one of their scientific aims and research priorities [9].

This study accessed plasma samples from healthy neonates through to adults and accordingly is the first study to investigate the physiological development of the human plasma proteome by examining the quantitative and qualitative changes that occur with age. The results provide an insight into the function of this complex biological fluid, as well as the processes of growth, development and ageing of the plasma proteome.

## Methods

### Sample information

Written informed consent was obtained and the study was approved by the Royal Children's Hospital Ethics in Human Research Committee (#20031E) and the Royal Women's Hospital Human Research Ethics Committee (#02/08). The consent was by provided by a parent or guardian for participants under the age of eighteen.

Six individual samples (3 males and 3 females) from each of the seven age-groups (Day1 neonates, Day 3 neonates; <1, 1–5, 6–10 and 11–16 years of age and adults) were used for this analysis.

Plasma samples were obtained from healthy children about to undergo elective surgery at the Royal Children's Hospital. These children were healthy other than the need for elective surgery (i.e. tongue tie release), were not receiving any medications and had no significant family history of major diseases, particularly diseases known to be associated with the process of ageing. Neonatal samples (day 1 and day 3 post-birth) were collected from healthy term neonates from the Family Birthing Unit or post-natal wards at the Royal Women's Hospital, Melbourne. Eligibility criteria included: gestation >37 weeks, vaginal delivery, birth-weight >2500 gm, APGAR at 5 min≥7 and absence of systemic abnormalities. Adults (21–43 years of age) were healthy volunteers on no medications, and similarly, with no past medical history.

Blood samples were collected in S-Monovette® tubes (Sarstedt, Australia), containing 1 volume of citrate per 9 volumes of blood and were centrifuged at 3000 rpm, 10 minutes, 10°C (Megafuge 1.0R, Heraeus), with plasma stored at −85°C, until testing.

Plasma samples were depleted of Albumin and IgG using the Albumin IgG removal kit (GE Healthcare, Rydalmere, Australia). The remaining proteins were precipitated using acetone precipitation, as specified in the depletion kit and were re-suspended in buffer containing 7 M urea, 2 M thiourea, 4% CHAPS and 30 mM Tris. Protein content of each sample was quantified using the Bradford assay (Bio-Rad, Hercules, CA, USA).

### 2D- Difference In Gel Electrophoresis (DIGE)

The internal standard consisted of an equal amount of each one of the 42 samples, was labeled with the Cyanine 2 (Cy2) dye (GE Healthcare, Rydalmere, Australia) and run on each gel to control for gel-to-gel variation. For each age-group, 3 samples were labeled with Cy3 and 3 samples with Cy5, with the samples then randomized to 42 gels run during this project (21 gels for pH 3–11; 21 gels for pH 4–7).

The Cy2, Cy3 and Cy5 samples (50 µg of sample/400 pmol of Cy dye) for each gel were pooled and loaded onto the 1^st^ Dimension Immobilized pH Gradient (IPG) Strip. The 24 cm, pH 3–11 and pH 4–7, non-linear Immobiline Drystrips (GE Healthcare, Rydalmere, Australia) were rehydrated with 15 µl IPG buffer 3–11/4–7 NL and 3 ml DeStreak solution (GE Healthcare, Rydalmere, Australia). Isoelectric focusing was carried out using the Multiphor II Isoelectric Focusing system (GE Healthcare, Rydalmere, Australia).

The IPG strips were conditioned for 15 min in equilibration buffer containing 2% Sodium Dodecyl Sulphate (SDS), 50 mM Tris(hydroxymethyl)aminomethane (Tris)-HCl, pH 8.8, 6 M urea, 30% glycerol, 0.002% bromophenol blue, and 10 mg/mL dithiothreitol (DTT). The strips were then alkylated for 15 min in equilibration buffer containing 25 mg/mL iodoacetamide instead of the DTT and loaded onto 12.5% polyacrylamide gels which were cast according to specifications by GE Healthcare. Second dimension was run using the Ettan Dalt 6 system (GE Healthcare, Rydalmere, Australia), for an average of 4.5 hours, until the bromophenol blue dye-front reached the edge of the gels.

### Gel Imaging

Gels were scanned using the Typhoon Trio variable mode imager (GE Healthcare, Rydalmere, Australia), with a resolution of 100 µm, and PMT of 500/600 V. Gels representing pH 3–11 were grouped and analyzed separately to gels representing pH 4–7.

### Gel Data Analysis

Data obtained from the gels was quantified using the DeCyder version 6.5 software (GE Healthcare, Rydalmere, Australia). The Differential In-gel analysis (DIA) was used to optimize spot detection. The Biological Variation Analysis (BVA) module was used for analysis of each sample according to the corresponding age-group. The filtering parameters were set to determine the spots that: had a p-value≤0.05 for the t-test (one way analysis of Variance ANOVA) employed to test the variation between age-groups and a>1.5 fold change in abundance between the groups.

### Spot excision and identification

Proteins of interest were excised from 2-D gels robotically using the Ettan Spot-picker (GE Healthcare, Rydalmere, Australia). Gel pieces were digested initially with 10 µL of 4 ng/µL endoprotease 1 (Lys-C) from *Achromobacter lyticus* (Wako Chemicals, Osaka, Japan) in 25 mM NH_4_HCO_3_, pH 9.0, overnight at 20°C and subsequently with 10 µL of 4 ng/µL sequencing grade porcine trypsin (Promega, Madison, WI, USA) in 25 mM NH_4_HCO_3_, pH 9.0 for 30 min at 37°C. The resulting peptides were acidified with 2 µL of 10% trifluoroacetic acid (TFA) and extracted by sonication. The peptides were purified using C18 reversed-phase ZipTips (Millipore Corp., Bedford, USA). Peptides were eluted with 5 µL 70% acetonitrile in 0.1% TFA for Matrix-assisted laser desorption/ionization – Time of Flight/Time of Flight (MALDI-TOF/TOF) analysis. A 0.5 µL sample aliquot was spotted onto a sample plate, which was pre-spotted with 0.5 µL of matrix (8 mg/mL α-cyano-4-hydroxycinnamic acid in 70% v/v acetonitrile and 0.1% TFA) and allowed to air dry. MALDI TOF/TOF analysis was performed with an Applied Biosystems 4800 Proteomics Analyser (Australian Cancer Research Foundation Biomolecular Resource Facility, The Australian National University, Canberra). The peak list-generating software used was AB MDS SCIEX 4000 Series Explorer version 3.5.1.

Spectra were acquired in Mass Spectrometry (MS) reflector mode over the m/z range of 800–3500 Da with internal calibration to trypsin autolytic peptides. The instrument was then switched to MS/MS mode where the 7 strongest peptides (excluding known contaminant peptides) from the MS scan were selected and fragmented using the Collision Induced Dissociation (CID) at low pressure and their mass and intensities were measured. A near point external calibration was applied for the MS/MS spectra and gave a typical mass accuracy of ∼50 ppm or less. Analysis of peptide data was performed using the Human International Protein Index version 3.49 and Protein Pilot version 2.0 (AB MDS SCIEX) databases. The number of protein entries searched was 74013, the cut-off score was >1.3 and the probability score of 95% that a protein is correct was used for accepting individual MS/MS spectra.

### Extended Data Analysis (EDA)

The EDA component of the DeCyder software (GE Healthcare, Rydalmere, Australia) was used for more specific analysis of the expression patterns of the plasma proteome proteins across the seven age-groups. Hierarchical clustering was used to group protein expression changes of differentially abundant proteins across the age-groups.

### Western blot validation

The validation of the protein abundance observed using 2D-DIGE was performed using western blotting. Pooled plasma samples containing equal amounts of protein from 6 individuals for each of the 7 age-groups were diluted 1∶100 using Phosphate Buffered Saline (PBS) and were run on 12% Tris-HCl SDS-Polyacrylamide Gel Electrophoresis (PAGE) gels (Bio-Rad, Australia) and transferred onto Amersham Hybond-P Polyvinylidene Fluoride (PVDF) membranes (GE Healthcare, Australia) using previously described method [10]. Membranes were immunoblotted with the sheep anti-human fibrinogen peroxidase conjugated IgG monoclonal antibody (SAFG-APHRP) or the goat anti-human alpha-2-macroglobulin peroxidase conjugated IgG monoclonal antibody (GAA2M-APHRP), at a dilution of 1∶10000 (Affinity Biologicals, Ontario, Canada). Fibrinogen and alpha-2-macroglobulin were visualized using the Electrochemiluminescence (ECL) Advance Western Blotting Detection Kit and images acquired using the Typhoon Trio variable mode imager, both from GE Healthcare. Quantification of the relative abundances was performed using the 1D gel analysis module from the ImageQuant TL version 7.0 software (GE Healthcare, Rydalmere, Australia).

### Phosphoproteome analysis

Age-related differences in phosphorylation of plasma proteins were determined by studying the plasma phosphoproteome for Day 1 neonates and Adults (N = 6 individual samples pooled for each group). Individual samples were depleted of Albumin and IgG, precipitated, re-suspended and quantified according to instructions above. 50 µg of sample from each of the two pools was used for the duplicate gel run. 2D-DIGE, image acquisition and analysis were performed according to the methods described earlier. Gels were stained with the Pro-Q Diamond phosphoprotein stain (Molecular Probes, Oregon, USA) according to instructions provided by the manufacturer. Pro-Q Diamond stained gel images were acquired using the Typhoon Trio – Variable mode imager (GE Healthcare, Rydalmere, Australia): excitation at 532 nm; emission filter-580 BP 30. Gel images were processed and matched to the master gel from the original workspace. Only proteins with known identities were considered. Cy5 (total protein) and Cy3 (quantity of phosphorylation) images and spot profiles of proteins were compared to determine the phosphorylation status. In the case of the protein being highly phosphorylated, the ratio of Cy5∶Cy3 spot volume is expected to be approximately 1.

## Results

### Protein spot maps and protein identity

**Figure 1:**
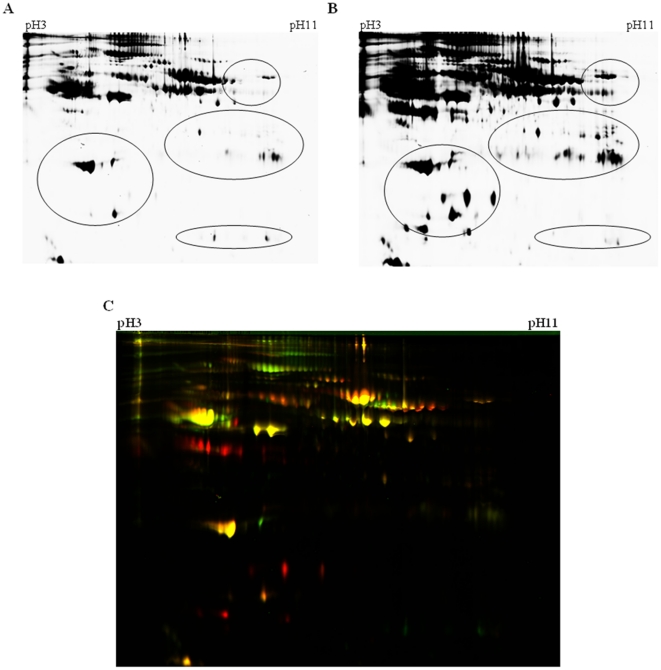
Comparison of the protein pattern from a representative 2D-DIGE gel of human plasma proteins. A: Cy3 stained Day 1 sample; B: Cy5 stained Adult sample; C: Image overlay (Cy3 - green, Cy5 - red, Identical areas – yellow). pH 3–11 left to right.

Approximately 1000 protein spots were detected on each gel. Up to 100 (10%) protein spots were found to be significantly different in abundance across various age-groups tested (Day 1 and Day 3 neonates, <1 year, 1–5, 6–10 and 11 to 16 year old children as well as adults) and occurred in more than 75% of the replicates. A representative spot pattern for a day 1 neonate and an adult sample are shown in Figure 1A and 1B respectively. Some of the obvious differences between the two samples are highlighted in both figures (circles), while the overlay in Figure 1C also highlights the differences between a plasma proteome for a Day 1 neonate and an Adult. The proteins that are less abundant in the neonatal sample are presented in red (Figure 1C) and the identified proteins are presented in Table 1; while the proteins that are more abundant in the neonatal sample are presented in green (Figure 1C) and the identified proteins are listed in Table 2. Some of the proteins that were present in neonatal plasma and absent from adult plasma and vice versa are as yet unidentifiable.

**Table 1:** Differentially expressed protein spots identified from the 2D-DIGE profiling of human plasma, with lower abundance in neonates and children compared to adults.

Protein	Master Number	Protein Name	Accession number	One way ANOVA	Protein Score	Protein Score C.I. %
1	608	Clusterin	P10909	2.92E-05	130	100
2	272	Isoform 2 of Fibrinogen alpha chain	P02671	4.80E-03	65	97.6
3	601	Isoform 2 of Fibrinogen alpha chain	P02671	2.21E-03	251	100
4	353	Isoform 2 of Fibrinogen alpha chain	P02671	0.0197	151	100
5	327	Isoform 2 of Fibrinogen alpha chain	P02671	0.0208	268	100
6	342	Isoform 2 of Fibrinogen alpha chain	P02671	0.0118	323	100
7	823	Fibrinogen beta chain	P02675	3.27E-03	139	100
8	477	Haptoglobin	P00738	1.85E-03	134	100
9	554	cDNA FLJ31310fis; Haptoglobin	B3KP77	8.07E-04	119	100
10	511	cDNA FLJ31310fis; Haptoglobin	B3KP77	2.04E-03	61	94.4
11	487	cDNA FLJ31310fis; Haptoglobin	B3KP77	2.53E-03	182	100
12	557	Haptoglobin isoform 2 preproprotein	P00739-2	1.37E-03	218	100
13	523	Haptoglobin isoform 2 preproprotein	P00739-2	1.95E-03	79	99.9
14	598	Isoform 1 of alpha-1-antitrypsin	P01009-1	1.37E-03	214	100
15	874	Hemopexin precursor	P02790	0.0016	71	98.273
16	825	Kininogen 1 Variant	P01042	0.004	111	100
17	866	Kininogen 1 Variant	P01042	0.0033	118	100
18	826	Kininogen 1 Variant	P10402	0.0062	82	99.869
19	855	Chain A, Cleaved Alpha1 -Antichymotrypsin	P01011	0.033	188	100
20	821	Hemopexin precursor	P02790	0.0078	236	100
21	277	Chain A, Human Factor H	P14210	0.048	428	100

Proteins were identified using MALTI-TOF/TOF. IPI – International Protein Index human accession number. Protein spots 1–14 were identified from pH 3–11 gels; Protein spots 15–21 were identified from pH 4–7 gels. Protein score reflects the combined scores of all observed mass spectra that can be matched to amino acid sequences within a specific protein; higher score indicates a more confident match.

**Table 2:** Differentially expressed protein spots identified from the 2D-DIGE profiling of human plasma, with higher abundance in neonates and children compared to adults.

Protein	Master Number	Name	Accession number	One way ANOVA	Protein Score	Protein Score C.I. %
1	100	Alpha-2-macroglobulin	P01023	1.37E-03	35	100
2	12	Alpha-2-macroglobulin	P01023	7.84E-03	251	100
3	149	Complement C3 (fragment)	P01024	9.08E-03	139	100
4	483	Complement C3	P01024	5.90E-03	298	100
5	333	Complement Factor B	P00751	5.48E-03	223	100
6	322	Isoform 2 of Fibrinogen alpha chain	P02671	4.06E-04	376	99.9
7	314	Isoform 2 of Fibrinogen alpha chain	P02671	5.41E-03	124	100
8	393	Fibrinogen beta chain	P02675	0.0113	153	100
9	335	Fibrinogen beta chain	P02675	3.86E-03	144	100
10	425	Isoform Gamma-A of Fibrinogen gamma chain	P02679	6.92E-03	114	100
11	345	Vitamin D-binding protein	P02774	4.14E-03	136	100
12	30	Fibronectin 1 variant	P02751	0.043	408	100
13	426	Alpha 2 macroglobulin variant	P01023	0.011	517	100
14	470	Chain B, Complement Component C3b	P01024	0.032	525	100
15	708	Chain A, Heparin Cofactor II	P05546	0.0087	147	100
16	940	Alpha-1-antichymotrypsin	P01011	0.012	183	100
17	416	Chain A, Bikunin From the Inter-Alpha Inhibitor Complex	P02760	0.04	88	99.963
18	427	Chain B, Complement Component C3	P01024	0.027	103	99.999
19	150	Alpha-2-macroglobulin	P01023	0.0077	91	99.984

Proteins were identified using MALTI-TOF/TOF. IPI – International Protein Index accession number. Protein spots 1–11 were identified from pH 3–11 gels; Protein spots 12–19 were identified from pH 4–7 gels. Protein score reflects the combined scores of all observed mass spectra that can be matched to amino acid sequences within a specific protein; higher score indicates a more confident match.

### Consideration of individual proteins

**Figure 2:**
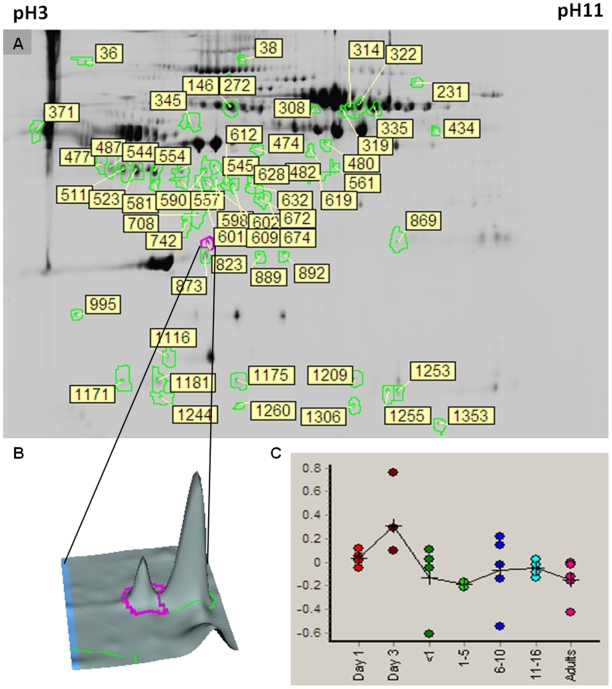
Example of protein that changes with age. (A) Representative gel image showing locations of proteins that are differentially expressed across age, ≥1.5 change in abundance; p<0.05; (B) 3-D view example of a spot that is up-regulated with age (number 823); (C) expression pattern for spot 823 (Fibrinogen beta chain) across seven age-groups; y-axis represents log abundance.

**Figure 3:**
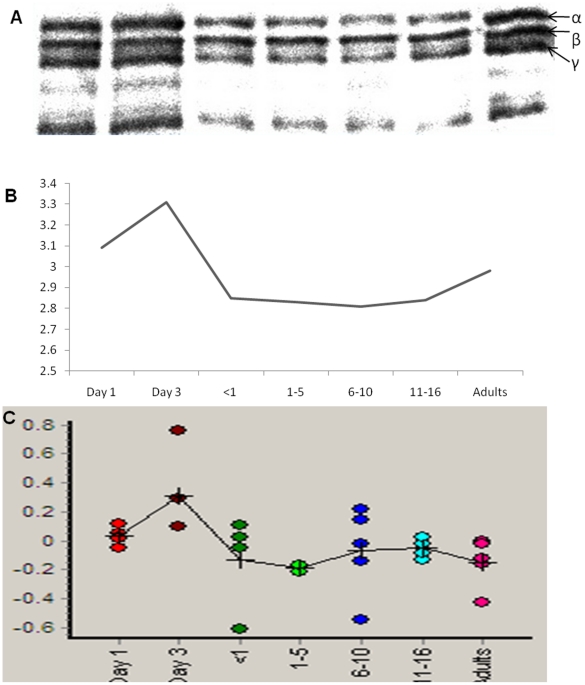
Validation of the age-specific variation in fibrinogen. (A) western blot image; (B) quantification (µg) of the western blot; (C) 2D-DIGE calculated relative log abundance.

**Figure 4:**
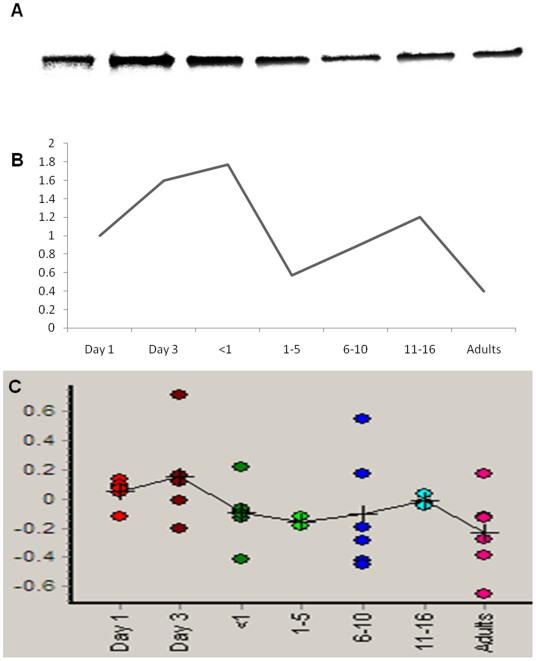
Validation of the age-specific variation in alpha-2-macroglobulin. (A) western blot image; (B) semi-quantification of the western blot (densitometry units); (C) 2D-DIGE calculated relative log abundance.

The location of proteins whose abundance changes significantly with age is shown in Figure 2A. An example of one such protein, fibrinogen beta chain, is shown in Figure 2B, with the age-related change in abundance for that protein demonstrated in Figure 2C. The age-specific abundance pattern for fibrinogen determined using the 2D-DIGE was validated using a fibrinogen specific antibody (Figure 3). The western blot validation was also performed for alpha-2-macroglobulin using a monoclonal antibody (Figure 4). In both cases, the age-related variation was consistent between the two methods employed.

### Gender specific analysis

The analysis of the protein abundance data based on gender of the participants demonstrates that 15 (0.87%) to 26 (1.8%) of the protein spots detected have significantly different abundance (1-ANOVA<0.05 and average ratio ≥1.5 or ≤−1.5) based on a gender analysis only, for pH 3–11 and pH 4–7 respectively.

### Proteins with common expression patterns

**Figure 5:**
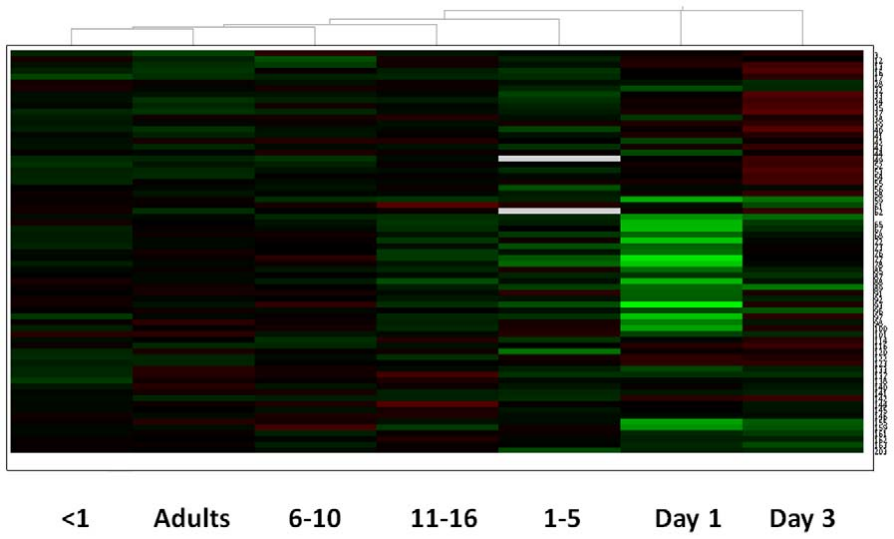
Hierarchical clustering of differentially abundant proteins across the age-groups. Relative expression patterns of each protein are expressed as a heat map ranging from −0.5 (green) to +0.5 (red), including proteins identified in Table 1 and Table 2.

The hierarchical clustering of the different age-groups, taking into account only the protein spots that were differentially abundant across all age-groups is represented in Figure 5. For the proteins that are significantly different in abundance across the seven age-groups, adults are closely related to the less than one year olds and that Day 1 and Day 3 neonates are closely linked to the 1 to 5 year old age-group.

**Figure 6:**
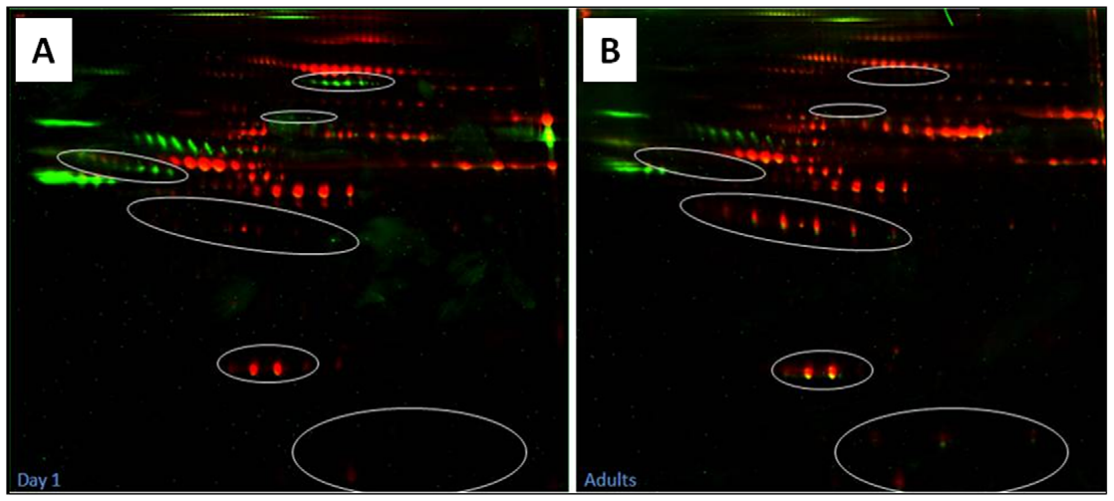
Overlay of gel stained with Pro-Q Diamond Phosphoprotein Stain. Circled areas represent regions with differences in phosphorylation patterns between (A) Day 1 neonates and (B) adult samples. Red (Cy5) represents the total plasma proteome; Green (Cy3) represents the phosphoproteome.

Differences in the phosphoproteome of neonatal and adult plasma are highlighted in Figure 6. From the plasma proteins with confirmed identification, 16 proteins were determined to be phosphorylated in each age-group (Table 3), with two proteins being specific to the neonatal group and an additional two proteins specific to the adult group. Based on the spot volume ratios (Cy5 (total)/Cy3 (phosphorylated)) proteins in the adult age-group were more phosphorylated compared to neonates.

**Table 3:** Phosphorylated proteins.

PROTEIN	Day1 Neonates	Adults
**Alpha-1-antichymotrypsin precursor**	**19.46**	**-**
Alpha-1-antichymotrypsin	3.04	1.03
Alpha-1B-glycoprotein	5.37	1.44
Antithrombin	6.28	2.27
Apo Serum Transferrin	9.54	1.37
Bikunin	3.61	−4.29
**Complement 3b**	**14.92**	**-**
Fibrinogen gamma	5.77	1.83
Haptoglobin	2.45	1.59
Hemopexin precursor	10.89	3.01
Inter-alpha (globulin) inhibitor H4	1.61	2.43
Kininogen	−9.62	−3.66
Prothrombin	4.79	1.25
Vitamin D Binding protein	12.36	3.9
**Alpha-1-antitrypsin precursor**	**-**	**6.01**
**Complement 9**	**-**	**3.04**

Results represent the spot volume ratio for total (Cy5) compared to phosphorylated (Cy3) protein. (-) Proteins that are not detectable in a particular age-group.

## Discussion

The results of this study provide important insights into plasma proteome changes accompanying normal growth and development. The differences in the plasma proteome with age are evident across the seven age-groups tested and for samples collected using specific inclusion criteria.

The sample size in our study is based on studies that investigated the plasma proteome [11], [12], as well as other proteomes [13], [14]. The carefully controlled selection of subjects in this study, as well as standardized blood collection, processing and storage protocol across all age-groups minimized the variation of external factors.

In addition to the age-specific differences, we confirm for the first time that up to 1.8% of the plasma proteome differences can be attributed to the gender of the participant. Importantly however, the proteins differentially expressed based on gender were not those proteins that were differentially expressed with age, leading to the conclusion that age-related changes in the plasma proteome are not a function of sexual maturity.

Some of the proteins contributing to this concept of “ageing” of the plasma proteome are known to be involved in iron transport and homeostasis, immune response, haemoglobin binding, serum protein transport, haemostasis, cholesterol metabolism, actin filament assembly and disassembly, clearance of cellular debris and apoptosis, regulation of muscle contraction as well as maintenance of osmotic pressure. This implies that the process of ageing is a multi-factorial, complex process that involves many processes which should be considered when studying diseases of ageing.

Post-translational modifications, particularly phosphorylation, may contribute to age-related changes in protein expression in plasma. This is the first study to demonstrate an age-related increase in protein phosphorylation in adult compared to neonatal plasma, suggesting that this difference in phosphorylation could be a function of age. Whether increased protein phosphorylation is in any way linked to an increasing prevalence of diseases in adults needs to be investigated further especially given the instrumental role of phosphorylation in modulating protein structure and function and the regulation of signalling pathways. It is particularly important to determine whether differences in the plasma phosphoproteome observed between neonates and adults persist throughout childhood.

The results of this study demonstrate a quantitative increase in fibrinogen in neonates (Day 1 and Day 3) compared to adults, a result that was validated using the western blot methodology. Fibrinogen plays an essential role in haemostasis, and is important for the formation of a stable clot. In addition, fibrinogen has also been shown to play an important role in the in the innate immune system, demonstrating the important interplay between hemostatic and inflammatory pathways [15]–[18]. Increased fibrinogen levels in neonates (Day 1 and Day 3) could play a role in the immune response that takes place immediately after birth. Age-specific reference ranges for fibrinogen are based on a functional assay, and although they demonstrate an increase in the functional activity of fibrinogen in infants and children up to 5 years of age [19], this is the first study to demonstrate age-related quantitative changes in fibrinogen. The fact that increased fibrinogen levels in neonates are not reflected by increased fibrinogen activity at this particular age, suggests a possible variation in the structure of this molecule in neonates and whether this could be a result of specific PTMs is still to be confirmed. This is certainly a relevant hypothesis, particularly with the knowledge that a “foetal” form of Fibrinogen exists in the first six month following birth, and has an increased sialic acid content compared to the adult form of the protein [20].

The abundance of alpha-2-macroglobulin was increased in neonates and children, compared to adults, a result which is consistent with previous evidence that alpha-2-macroglobulin quantity decreases with age [21]. Since, the incidence of thromboembolic events is significantly decreased in healthy neonates and children compared to adults, the increased expression of alpha-2-macroglobulin in early age may be one of the factors contributing to the overall thromboprotective state observed in childhood. Furthermore, the decline in alpha-2-macroglobulin with increasing age could be explained by the genetic association between alpha-2-macroglobulin polymorphisms in Alzheimer's disease. Specifically, the underlying assumption is that the (5 base pair intronic) deletion in the Alpha-2-macroglobulin gene may affect the functionality and quantity of the resultant Alpha-2-macroglobulin protein in circulation, therefore contributing to Alzheimer's disease pathology [22]. Alternatively, it is possible that neither genetic predisposition nor Alpha-2-macroglobulin quantity contribute towards the pathology of Alzheimer's disease, but rather differences in post-translational modification of this protein. Taking into account that there is currently no cure available to treat this incapacitating disease, the observed age-related decrease in abundance of alpha-2-macroglobulin with advancing age might prove to be relevant to Alzheimer's disease pathology and thus provides a potential focus for future research.

The increased expression of plasma Alpha-1-antichymotrypsin in neonates and children compared to adults is a novel finding, as age-specific reference ranges for Alpha-1-antichymotrypsin in neonates and children have not been reported to date. Alpha-1-antichymotrypsin deficient plasma (due to a point mutation in the Alpha-1-antichymotrypsin gene) is associated with a predisposition to chronic pulmonary obstructive disorders [23], [24]. This is confirmed by Alpha-1-antichymotrypsin's role as a protease inhibitor in the defence against degradation by proteases in the lower respiratory airways [25]. Therefore, observed age-related decrease in plasma Alpha-1-antichymotrypsin in the current study is consistent with the increased prevalence of chronic obstructive pulmonary disorders in the adults compared to neonates and children [26]. Whether this decrease in Alpha-1-antichymotrypsin with age may be a causative or an associated phenomenon with disease should be determined with further studies.

The abundance of Kininogen was increased in adults compared to neonates and children. This finding that the abundance of Kininogen increases with age is consistent with the knowledge that elevated levels of plasma Kininogen are associated with diseases of ageing, such as vascular disease and thromboembolism [27], rheumatoid disease [28], Paget's disease [28] and also with increased risk of myocardial infarction [29]. Whether increased levels of Kininogen may prove to be a potential marker for detection of certain diseases remains to be determined.

Increased expression of the vitamin D binding protein in neonates and children compared to adults is novel and important, particularly with the knowledge that higher vitamin D circulating concentrations are associated with a lower risk of chronic illnesses such as common cancers as well as cardiac diseases [30].

Another novel finding of this study relates to clusterin, which we show to have decreased expression in neonates and children compared to adults. The expression of clusterin, also known as apolipoprotein J is known to be induced by processes such as oxidative stress, as well as apoptotic stimuli and this protein has also been implicated in inhibition of neuroblastoma cell invasion [31]. Considering that these processes are stimulated by ageing, our observation of age-related increase in abundance of clusterin confirms the relevance of this protein in the process of ageing and suggests another avenue for future research.

The results of the cluster analysis seem to be counter-intuitive, with Day 1 and Day 3 neonates linking more closely to the 1–5 year old age-group, rather than to the <1 year old age-group. However, these results are based only on the differentially abundant proteins, not the detected plasma proteome as a whole and demonstrate that the neonatal age-groups in general have more proteins that are found in lower abundance compared to other age-groups.

The potential limitations of the study include the number of the samples tested, as well as the fact that the method chosen is one of a number of methods available at present, all of which do not represent the whole, but only a part of the plasma proteome. In the future, methods might become available to investigate the complete plasma proteome. However, this is not a viable option at present.

Despite these limitations, our study suggests age-related changes in protein abundance and phosphorylation is common amongst a number of plasma proteins. The functional implications of these changes remains to be determined and likely has major implications for our understanding of the role of these proteins in a wide range of physiological functions during growth and development. In addition, given that the incidence of the majority of diseases (i.e. diabetes, cardiovascular disease, cancer) increase with age, this study provides the basis for identification of potential biomarkers and therapeutic targets for numerous disease processes.

By understanding the age-specific development of the plasma proteome, future investigations can focus on disease related processes in this complex biological fluid.
